# Can electrical stimulation of the human bed nucleus of the stria terminalis reduce contextual anxiety? An unanswered question

**DOI:** 10.3389/fnbeh.2015.00069

**Published:** 2015-03-17

**Authors:** Kelly Luyck, Laura Luyten

**Affiliations:** ^1^Experimental Neurosurgery and Neuroanatomy, Department of Neurosciences, KU LeuvenLeuven, Belgium; ^2^Psychology of Learning and Experimental Psychopathology, Psychology and Educational Sciences, KU LeuvenLeuven, Belgium

**Keywords:** deep brain stimulation, obsessive–compulsive disorder, fear-potentiated startle, context, bed nucleus of the stria terminalis, anterior limb of the internal capsule, anxiety, context conditioning

In their paper, Baas et al. (2014), take the rather unique opportunity to conduct a human fear conditioning experiment in subjects with stimulation electrodes in the nucleus accumbens and internal capsule. Here, we argue that it might be premature to draw the strong conclusion that deep brain stimulation (DBS) does not impact contextual anxiety. We will elaborate on two aspects of the study that were not thoroughly discussed in the original paper, but that might help explain why no effects were found. Firstly, we will focus on the neuroanatomy and, secondly, on the behavioral procedure.

The authors did not mention the precise location of the stimulation contact, but previous work from the same group allowed us to piece together a more detailed view of the actual stimulation site. While the distal lead tip was implanted in the nucleus accumbens, the active contact was located in the ventral part of the anterior limb of the internal capsule (ALIC) (van den Munckhof et al., 2013). The authors stated that these active contacts overlap with the bed nucleus of the stria terminalis (BST), or are at least in close proximity to this nucleus. Moreover, they hypothesized that they would be able to stimulate the BST and/or its connections and thereby affect contextual anxiety in their patients. However, a closer examination of the neuroanatomy shows that it is unlikely that the authors could sufficiently influence the BST (Figure 1). With monopolar stimulation, it can be estimated that the stimulated volume extends 4.4 mm (the average stimulation amplitude used in these patients was 4.4 V) from the center of the electrode (Mädler and Coenen, 2012). The anterior tip of the BST is located 3.4 mm from the active contact, in theory allowing the researchers to stimulate the most anterior millimeter of the BST (which has a total anterior-posterior extent of about 6.5 mm). Importantly, it appears that this most anterior BST portion is not the essential location with regard to (unpredictable) threat. Rather, it seems that more posterior parts of the BST, further away from the contact and outside of the stimulated volume, are involved in human threat paradigms (Figure 1) (Straube et al., 2007; Somerville et al., 2010; Alvarez et al., 2011). In line with this, we previously found that BST lesions clearly reduced startle potentiation in a rat context conditioning paradigm, but more anterior lesions (closer to the target of Baas et al., 2014) did not (Luyten et al., 2011).

**Figure 1:**
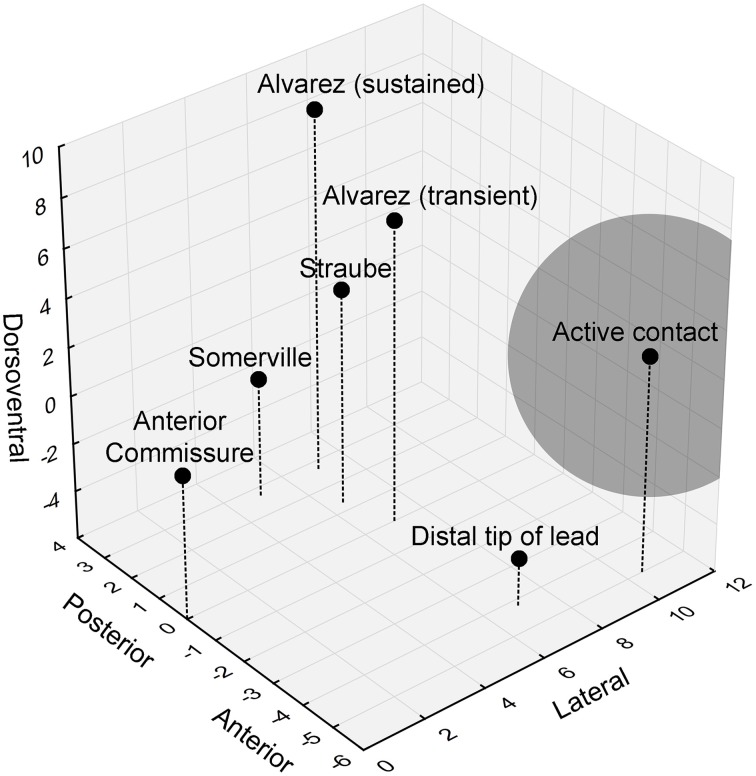
**3D visualization of the relevant anatomical locations**. Black dots indicate the positions of the anterior commissure, the distal lead tip and active contact in Baas et al. (2014), and the peak hyperactive BST subregions in previous studies looking at threat activation of the BST. The gray sphere indicates the estimated stimulated volume (about 4.4 mm radiating from the center of the electrode). Coordinates (in mm) are based upon (Mai et al., 2008).

Although we do not exclude that some fibers in the internal capsule might project to (the bed nucleus of) the stria terminalis, it is clear that the ALIC is a complex collection of fibers with various destinations (Lehman et al., 2011). By stimulating this fiber bundle, or, by analogy, calling several telephone lines at once, it is uncertain who will answer the phone. Targets of adjacent fibers might even have opposite functions (Rodriguez-Romaguera et al., 2012), thereby canceling out a behavioral effect. Therefore, it is probably a stretch to assume that one can specifically manipulate BST activity through ALIC stimulation. Taken together, it seems highly unlikely that the authors could precisely and sufficiently influence relevant parts of the BST, given the location of their stimulation contacts.

Note that, if one would actually stimulate the human BST, this might produce various, and even contrasting effects, such as an overall decrease of anxious mood and associated somatic symptoms (as indexed by the Hamilton Anxiety Rating Scale in the DBS for OCD study Baas et al., 2014), but no effect on anxiety tests with a more specific scope. In this regard, it is important to keep in mind that the BST is an extremely complex structure involved in various autonomic, neuroendocrine and behavioral responses (Dumont, 2009). Neuroanatomically, we can distinguish numerous subdivisions (Ju and Swanson, 1989; Moga et al., 1989; Alheid et al., 1998) with divergent neurophysiological characteristics (Haufler et al., 2013; Rodriguez-Sierra et al., 2013) and connections (Weller and Smith, 1982; Dong and Swanson, 2004, 2006). Even though there is convincing evidence from local lesion or inactivation experiments (Hitchcock and Davis, 1991; Lee and Davis, 1997; Sullivan et al., 2004) and imaging studies (Alvarez et al., 2011; Luyten et al., 2012) that the BST mediates sustained anxiety responses, earlier papers already suggested that BST subdivisions may have different, sometimes even opposing functions. For instance, electrical stimulation of the medial part of the BST increased plasma corticosterone levels, while stimulation of the lateral part had the opposite effect (Dunn, 1987). Similarly, electrical stimulation of the medial BST was found to increase blood pressure and heart rate, whereas stimulation of the lateral part decreased these cardiovascular parameters (Dunn and Williams, 1995).

Recently, major progress has been made in untangling the neuronal circuits of the BST using optogenetics, which allows for very precise manipulation of specific neuronal populations (Deisseroth, 2011; Johansen, 2013). Kim et al. found that photostimulation of the basolateral amygdala inputs into the anterodorsal BST in mice resulted in anxiolytic effects (Kim et al., 2013). Optogenetic activation of the oval nucleus of the BST, which is surrounded by the anterodorsal part, was anxiogenic, as indicated by a decrease in time spent in the open arms of the elevated plus maze. Activation of the dorsal BST as a whole, including both the oval and anterodorsal parts, was found to be anxiogenic as well. In addition, Jennings et al. demonstrated that photostimulation of the glutamatergic connections between the ventral BST and ventral tegmental area was anxiogenic, whereas activation of the GABAergic projections in the same area resulted in anxiolytic effects (Jennings et al., 2013).

The optogenetic approach will undoubtedly continue to deepen our understanding of the BST, as it enables us to parcel out the roles of certain subdivisions or even cell types in the BST (Sparta et al., 2013). This, however, does not mean that research using long-standing techniques such as lesioning or electrical stimulation is redundant, especially given the clinical relevance of these methods for the treatment of psychiatric disorders with capsulotomy (Gabriëls et al., 2008; Lopes et al., 2014) or DBS (Denys et al., 2010; Greenberg et al., 2010). In fact, it might be interesting to combine these “old” and “new” techniques, as this may shed light on the poorly understood mechanisms of DBS (Fenoy et al., 2014; Okun, 2014). Finally, we should keep in mind that, although the overall BST neurocircuitry in humans and other animals is largely analogous (Avery et al., 2014), it remains to be seen if and how these recent optogenetic and neurophysiological findings can be extrapolated to the human BST.

Apart from these neuroanatomical concerns, we feel that Baas and colleagues could have increased their chance of finding an effect of DBS on context conditioning by promoting the presumed BST activation with their behavioral paradigm. Most evidence for a role of the BST in contextual anxiety comes from animal research (Sullivan et al., 2004; Luyten et al., 2012; Sink et al., 2013). To increase the translatability of these animal data, and therefore the chance of finding an effect of BST manipulation in human subjects, it seems advisable to maximize the comparability of both paradigms. Here, we will discuss three aspects of the authors' paradigm that differ from the rodent literature: the low complexity of the contexts, the use of instructed threat, and the intertwined acquisition and test phases.

In the real world, anxiety-evoking events occur in a complex environment that consists out of multiple stimuli and engages all our sensory modalities (Huff et al., 2011). For instance, war veterans suffering from post-traumatic stress disorder may be reminded of their traumatic experience by loud noises, the smell of smoke, the sight of a helicopter, etc. Likewise, in animal research, subjects are usually completely immersed in a physical context. In human fear conditioning, virtual reality is a useful tool to mimic these animal studies, as well as the complexity of real-life experiences (Baas et al., 2004). Notably, a key study that found human BST activation in response to unpredictable threat (Alvarez et al., 2011) used a virtual reality conditioning paradigm, with different environments through which the participants were passively guided, requiring complex contextual processing. In contrast, the current study used very simple context representations. Participants were given written instructions on a computer screen regarding the context they were in and the course of shock administration. We do not claim that a complex context is necessary for BST involvement, but it is an aspect that distinguishes the paradigm of Baas et al. (2014) from studies finding BST activation, both in animals and humans. Note that other groups have even proposed fully immersive, 3D virtual reality to bridge animal models of context conditioning and real-life human anxiety to a greater extent (Huff et al., 2011).

As mentioned above, Baas et al. (2014) provided clear information about shock likelihood and contingencies, using instructions that remained on the screen during the entire test session. This approach might rely less on experiential learning than other human fear conditioning studies (Fonteyne et al., 2009) and, more importantly, than animal conditioning studies of BST involvement. Interestingly, there are some preliminary indications that instructed vs. classical conditioning might have divergent neural correlates (Klucken et al., 2009; Mechias et al., 2010).

Regarding the timing of acquisition and expression, it is noteworthy that in rodent context conditioning, training and testing usually take place on 2 days. Due to practical concerns, however, human conditioning experiments are often carried out in one session (but see Huff et al., 2011; Sevenster et al., 2012). This may lead to confounded results, since acquisition still takes place when the expression of fear/anxiety is being measured. Furthermore, long-term processes including protein synthesis are required for consolidation of fear conditioning (Hoeffer et al., 2011), favoring the use of a 2-day protocol. Finally, we believe that a stronger temporal distinction between training and testing can be particularly valuable when exploring the therapeutic mechanisms of DBS. Since DBS is a curative rather than a preventive technique, it will mainly affect expression and not acquisition of patients' deep-rooted anxieties. Therefore, a useful approach would be to stimulate only during the testing phase of a human fear conditioning paradigm, without interfering with acquisition.

## Conflict of interest statement

We acknowledge the financial support of the Medtronic Chair for Stereotactic Neurosurgery in Psychiatric Disorders at KU Leuven.

## References

[B1] Alheid G. F. Beltramino C. A. De Olmos J. S. Forbes M. S. Swanson D. J. Heimer L. (1998). The neuronal organization of the supracapsular part of the stria terminalis in the rat: the dorsal component of the extended amygdala. Neuroscience 84, 967–996. 10.1016/S0306-4522(97)00560-5 9578390

[B2] Alvarez R. P. Chen G. Bodurka J. Kaplan R. Grillon C. (2011). Phasic and sustained fear in humans elicits distinct patterns of brain activity. Neuroimage 55, 389–400. 10.1016/j.neuroimage.2010.11.057 21111828 PMC3100535

[B3] Avery S. N. Clauss J. A. Winder D. G. Woodward N. Heckers S. Blackford J. U. (2014). BNST neurocircuitry in humans. Neuroimage 91, 311–323. 10.1016/j.neuroimage.2014.01.017 24444996 PMC4214684

[B4] Baas J. M. Klumpers F. Mantione M. H. Figee M. Vulink N. C. Schuurman P. R.. (2014). No impact of deep brain stimulation on fear-potentiated startle in obsessive-compulsive disorder. Front. Behav. Neurosci. 8:305. 10.3389/fnbeh.2014.00305 25249953 PMC4158815

[B5] Baas J. M. Nugent M. Lissek S. Pine D. S. Grillon C. (2004). Fear conditioning in virtual reality contexts: a new tool for the study of anxiety. Biol. Psychiatry 55, 1056–1060. 10.1016/j.biopsych.2004.02.024 15158423

[B6] Deisseroth K. (2011). Optogenetics. Nat. Methods 8, 26–29. 10.1038/nmeth.f.324 21191368 PMC6814250

[B7] Denys D. Mantione M. Figee M. van den Munckhof P. Koerselman F. Westenberg H.. (2010). Deep brain stimulation of the nucleus accumbens for treatment-refractory obsessive-compulsive disorder. Arch. Gen. Psychiatry 67, 1061–1068. 10.1001/archgenpsychiatry.2010.122 20921122

[B8] Dong H. W. Swanson L. W. (2004). Projections from bed nuclei of the stria terminalis, posterior division: implications for cerebral hemisphere regulation of defensive and reproductive behaviors. J. Comp. Neurol. 471, 396–433. 10.1002/cne.20002 15022261

[B9] Dong H. W. Swanson L. W. (2006). Projections from bed nuclei of the stria terminalis, anteromedial area: cerebral hemisphere integration of neuroendocrine, autonomic, and behavioral aspects of energy balance. J. Comp. Neurol. 494, 142–178. 10.1002/cne.20788 16304685 PMC2563961

[B10] Dumont E. C. (2009). What is the bed nucleus of the stria terminalis? Prog. Neuropsychopharmacol. Biol. Psychiatry 33, 1289–1290. 10.1016/j.pnpbp.2009.07.006 19602427 PMC4011829

[B11] Dunn J. D. (1987). Plasma corticosterone responses to electrical stimulation of the bed nucleus of the stria terminalis. Brain Res. 407, 327–331. 10.1016/0006-8993(87)91111-5 3567648

[B12] Dunn J. D. Williams T. J. (1995). Cardiovascular responses to electrical stimulation of the bed nucleus of the stria terminalis. J. Comp. Neurol. 352, 227–234. 10.1002/cne.903520206 7721991

[B13] Fenoy A. J. Goetz L. Chabardes S. Xia Y. (2014). Deep brain stimulation: are astrocytes a key driver behind the scene? CNS Neurosci. Ther. 20, 191–201. 10.1111/cns.12223 24456263 PMC3969941

[B14] Fonteyne R. Vervliet B. Hermans D. Baeyens F. Vansteenwegen D. (2009). Reducing chronic anxiety by making the threatening event predictable: an experimental approach. Behav. Res. Ther. 47, 830–839. 10.1016/j.brat.2009.06.011 19604499

[B15] Gabriëls L. Nuttin B. Cosyns P. (2008). Applicants for stereotactic neurosurgery for psychiatric disorders: role of the Flemish advisory board. Acta Psychiatr. Scand. 117, 381–389. 10.1111/j.1600-0447.2008.01166.x 18331579

[B16] Greenberg B. D. Gabriëls L. A. Malone D. A. Jr. Rezai A. R. Friehs G. M. Okun M. S.. (2010). Deep brain stimulation of the ventral internal capsule/ventral striatum for obsessive-compulsive disorder: worldwide experience. Mol. Psychiatry 15, 64–79. 10.1038/mp.2008.55 18490925 PMC3790898

[B17] Haufler D. Nagy F. Z. Pare D. (2013). Neuronal correlates of fear conditioning in the bed nucleus of the stria terminalis. Learn. Mem. 20, 633–641. 10.1101/lm.031799.113 24131794 PMC3799415

[B18] Hitchcock J. M. Davis M. (1991). Efferent pathway of the amygdala involved in conditioned fear as measured with the fear-potentiated startle paradigm. Behav. Neurosci. 105, 826–842. 10.1037/0735-7044.105.6.826 1663757

[B19] Hoeffer C. A. Cowansage K. K. Arnold E. C. Banko J. L. Moerke N. J. Rodriguez R.. (2011). Inhibition of the interactions between eukaryotic initiation factors 4E and 4G impairs long-term associative memory consolidation but not reconsolidation. Proc. Natl. Acad. Sci. U.S.A. 108, 3383–3388. 10.1073/pnas.1013063108 21289279 PMC3044415

[B20] Huff N. C. Hernandez J. A. Fecteau M. E. Zielinski D. J. Brady R. Labar K. S. (2011). Revealing context-specific conditioned fear memories with full immersion virtual reality. Front. Behav. Neurosci. 5:75. 10.3389/fnbeh.2011.00075 22069384 PMC3209582

[B21] Jennings J. H. Sparta D. R. Stamatakis A. M. Ung R. L. Pleil K. E. Kash T. L.. (2013). Distinct extended amygdala circuits for divergent motivational states. Nature 496, 224–228. 10.1038/nature12041 23515155 PMC3778934

[B22] Johansen J. P. (2013). Neuroscience: anxiety is the sum of its parts. Nature 496, 174–175. 10.1038/nature12087 23515160

[B23] Ju G. Swanson L. W. (1989). Studies on the cellular architecture of the bed nuclei of the stria terminalis in the rat: I. Cytoarchitecture. J. Comp. Neurol. 280, 587–602. 10.1002/cne.902800409 2708568

[B23a] Kim S.-Y. Adhikari A. Lee S. Y. Marshel J. H. Kim C. K. Mallory C. S.. (2013). Diverging neural pathways assemble a behavioural state from separable features in anxiety. Nature 496, 219–223. 10.1038/nature12018 23515158 PMC6690364

[B24] Klucken T. Tabbert K. Schweckendiek J. Merz C. J. Kagerer S. Vaitl D.. (2009). Contingency learning in human fear conditioning involves the ventral striatum. Hum. Brain Mapp. 30, 3636–3644. 10.1002/hbm.20791 19384886 PMC6871066

[B25] Lee Y. Davis M. (1997). Role of the hippocampus, the bed nucleus of the stria terminalis, and the amygdala in the excitatory effect of corticotropin-releasing hormone on the acoustic startle reflex. J. Neurosci. 17, 6434–6446. 9236251 10.1523/JNEUROSCI.17-16-06434.1997 PMC6568348

[B26] Lehman J. F. Greenberg B. D. McIntyre C. C. Rasmussen S. A. Haber S. N. (2011). Rules ventral prefrontal cortical axons use to reach their targets: implications for diffusion tensor imaging tractography and deep brain stimulation for psychiatric illness. J. Neurosci. 31, 10392–10402. 10.1523/JNEUROSCI.0595-11.2011 21753016 PMC3445013

[B27] Lopes A. C. Greenberg B. D. Canteras M. M. Batistuzzo M. C. Hoexter M. Q. Gentil A. F.. (2014). Gamma ventral capsulotomy for obsessive-compulsive disorder: a randomized clinical trial. JAMA Psychiatry 71, 1066–1076. 10.1001/jamapsychiatry.2014.1193 25054836

[B28] Luyten L. Casteels C. Vansteenwegen D. van Kuyck K. Koole M. Van Laere K.. (2012). Micro-positron emission tomography imaging of rat brain metabolism during expression of contextual conditioning. J. Neurosci. 32, 254–263. 10.1523/JNEUROSCI.3701-11.2012 22219287 PMC6621336

[B29] Luyten L. van Kuyck K. Vansteenwegen D. Nuttin B. (2011). Electrolytic lesions of the bed nucleus of the stria terminalis disrupt freezing and startle potentiation in a conditioned context. Behav. Brain Res. 222, 357–362. 10.1016/j.bbr.2011.03.066 21497171

[B30] Mädler B. Coenen V. A. (2012). Explaining clinical effects of deep brain stimulation through simplified target-specific modeling of the volume of activated tissue. Am. J. Neuroradiol. 33, 1072–1080. 10.3174/ajnr.A2906 22300931 PMC8013266

[B31] Mai J. K. Voss T. Paxinos G. (2008). Atlas of the Human Brain. London: Elsevier/Academic Press.

[B32] Mechias M. L. Etkin A. Kalisch R. (2010). A meta-analysis of instructed fear studies: implications for conscious appraisal of threat. Neuroimage 49, 1760–1768. 10.1016/j.neuroimage.2009.09.040 19786103

[B33] Moga M. M. Saper C. B. Gray T. S. (1989). Bed nucleus of the stria terminalis: cytoarchitecture, immunohistochemistry, and projection to the parabrachial nucleus in the rat. J. Comp. Neurol. 283, 315–332. 10.1002/cne.902830302 2568370

[B34] Okun M. S. (2014). Deep-brain stimulation–entering the era of human neural-network modulation. N. Engl. J. Med. 371, 1369–1373. 10.1056/NEJMp1408779 25197963

[B35] Rodriguez-Romaguera J. Do Monte F. H. Quirk G. J. (2012). Deep brain stimulation of the ventral striatum enhances extinction of conditioned fear. Proc. Natl. Acad. Sci. U.S.A. 109, 8764–8769. 10.1073/pnas.1200782109 22586125 PMC3365168

[B36] Rodriguez-Sierra O. E. Turesson H. K. Pare D. (2013). Contrasting distribution of physiological cell types in different regions of the bed nucleus of the stria terminalis. J. Neurophysiol. 110, 2037–2049. 10.1152/jn.00408.2013 23926040 PMC3841931

[B37] Sevenster D. Beckers T. Kindt M. (2012). Instructed extinction differentially affects the emotional and cognitive expression of associative fear memory. Psychophysiology 49, 1426–1435. 10.1111/j.1469-8986.2012.01450.x 22958209

[B38] Sink K. S. Davis M. Walker D. L. (2013). CGRP antagonist infused into the bed nucleus of the stria terminalis impairs the acquisition and expression of context but not discretely cued fear. Learn. Mem. 20, 730–739. 10.1101/lm.032482.113 24255102 PMC3834624

[B39] Somerville L. H. Whalen P. J. Kelley W. M. (2010). Human bed nucleus of the stria terminalis indexes hypervigilant threat monitoring. Biol. Psychiatry 68, 416–424. 10.1016/j.biopsych.2010.04.002 20497902 PMC2921460

[B40] Sparta D. R. Jennings J. H. Ung R. L. Stuber G. D. (2013). Optogenetic strategies to investigate neural circuitry engaged by stress. Behav. Brain Res. 255, 19–25. 10.1016/j.bbr.2013.05.007 23684554 PMC4415682

[B41] Straube T. Mentzel H. J. Miltner W. H. (2007). Waiting for spiders: brain activation during anticipatory anxiety in spider phobics. Neuroimage 37, 1427–1436. 10.1016/j.neuroimage.2007.06.023 17681799

[B42] Sullivan G. M. Apergis J. Bush D. E. Johnson L. R. Hou M. Ledoux J. E. (2004). Lesions in the bed nucleus of the stria terminalis disrupt corticosterone and freezing responses elicited by a contextual but not by a specific cue-conditioned fear stimulus. Neuroscience 128, 7–14. 10.1016/j.neuroscience.2004.06.015 15450349

[B43] van den Munckhof P. Bosch D. A. Mantione M. H. Figee M. Denys D. A. Schuurman P. R. (2013). Active stimulation site of nucleus accumbens deep brain stimulation in obsessive-compulsive disorder is localized in the ventral internal capsule. Acta Neurochirurgica. Suppl. 117, 53–59. 10.1007/978-3-7091-1482-7_9 23652657

[B44] Weller K. L. Smith D. A. (1982). Afferent connections to the bed nucleus of the stria terminalis. Brain Res. 232, 255–270. 10.1016/0006-8993(82)90272-4 7188024

